# Incidence and risk factors for non-union of the superior ramus osteotomy when hip dysplasia is treated with periacetabular osteotomy

**DOI:** 10.1093/jhps/hnad006

**Published:** 2023-04-12

**Authors:** Ganesh Sivamurugan, Robert W Westermann, Natalie Glass, John C Davison, Aspen Miller, Jacob Henrichsen, Todd O McKinley, Michael C Willey

**Affiliations:** Department of Orthopaedic Surgery and Rehabilitation, University of Iowa Hospitals and Clinics, 200 Hawkins Drive, Iowa City, IA 52242, USA; Department of Orthopaedic Surgery and Rehabilitation, University of Iowa Hospitals and Clinics, 200 Hawkins Drive, Iowa City, IA 52242, USA; Department of Orthopaedic Surgery and Rehabilitation, University of Iowa Hospitals and Clinics, 200 Hawkins Drive, Iowa City, IA 52242, USA; Department of Orthopaedic Surgery and Rehabilitation, University of Iowa Hospitals and Clinics, 200 Hawkins Drive, Iowa City, IA 52242, USA; Department of Orthopaedic Surgery and Rehabilitation, University of Iowa Hospitals and Clinics, 200 Hawkins Drive, Iowa City, IA 52242, USA; Department of Orthopaedic Surgery and Rehabilitation, University of Iowa Hospitals and Clinics, 200 Hawkins Drive, Iowa City, IA 52242, USA; Indiana University Health, Methodist Hospital, 1801 N Senate Boulevard, Suite 535, Indianapolis, IN 46202, USA; Department of Orthopaedic Surgery and Rehabilitation, University of Iowa Hospitals and Clinics, 200 Hawkins Drive, Iowa City, IA 52242, USA

## Abstract

Periacetabular osteotomy (PAO) is a well-established surgical treatment for hip dysplasia. Few studies report risk factors for the development of superior ramus osteotomy non-union. The purpose of this investigation was to document the incidence and risk factors for this complication. We identified 316 consecutive hips that underwent PAO for symptomatic acetabular dysplasia with a minimum 1-year radiographic follow-up. We developed and validated a technique to measure the superior ramus osteotomy location on anterior-posterior (AP) pelvis radiographs and computed tomography. Logistic regression with generalized estimating equations was used to evaluate the relationships between odds of non-union and potential demographic and radiographic predictor variables in univariate and multivariate analyses. Twenty-nine (9.2%) hips developed superior ramus non-union. Age {median [interquartile range (IQR)] 23 years (18–35) healed versus 35 years (26–40) non-united, *P* = 0.001}, pre-operative lateral center-edge angle (LCEA) [16° (11–20) healed versus 10° (6–13) non-united, *P* < 0.001] and the distance from the superior ramus osteotomy to the ilioishial line [15.8 mm (13.2–18.7) healed versus 18.1 mm (16.2–20.5) non-united, *P* < 0.001] varied significantly between groups. Using multivariate analysis, moderate-to-severe dysplasia [LCEA < 15°, odds ratio (OR) 5.95, standard error (SE) 3.32, 95% confidence interval (CI) 1.99–17.79, *P* = 0.001], increased age (5-year increase, OR 1.29, SE 3.32, 95% CI 1.105–1.60, *P*-value = 0.018) and distance from the ilioishial line (3-mm increase, OR 1.67, SE 0.22, 95% CI 1.29–2.18, *P* < 0.001) were at increased risk of developing non-union. Superior ramus osteotomy non-union is common after PAO. Older age, moderate-to-severe dysplasia, and more medial osteotomy location were independent risk factors for non-union. Consideration should be made in high-risk patients for a more lateral superior ramus osteotomy and adjuvant medical and surgical interventions.

## INTRODUCTION

Hip dysplasia is the most common cause of osteoarthritis in young adults [1]. Periacetabular osteotomy (PAO) is a well-established procedure to treat symptomatic acetabular dysplasia [2–9] but is technically demanding with a significant learning curve [10, 11]. Reported surgical complications include non-union of osteotomy sites, deep vein thrombosis, peroneal nerve dysfunction, heterotopic ossification, posterior column fracture, and intra-articular osteotomies [2–9]. One of the most commonly reported complications is superior ramus non-union with an incidence ranging from 1% to 17% [12–17]. Non-union of the superior ramus increases the risk of ischial stress fracture [17–19] and may require a secondary intervention to facilitate healing.

Further investigation is needed into the incidence and modifiable risk factors for superior ramus non-union when hip dysplasia is treated with PAO. It has been suggested that higher body mass index (BMI), older age, and more severe dysplasia increase the risk of non-union [12–16]. The impact of osteotomy location on the superior ramus has not been investigated as a risk factor for non-union. With the majority of the literature reporting the risk for superior ramus non-union as >5% [12–14], documenting modifiable risk factors for non-union would guide surgical technique and identify patients who may benefit from an adjuvant intervention to facilitate healing.

The purpose of this investigation was to (i) determine the incidence of superior pubic ramus non-union at a minimum 1-year radiographic follow-up after PAO to treat hip dysplasia, (ii) identify patient-specific risk factors associated with non-union of the superior pubic ramus, and (iii) determine if the position of the superior ramus osteotomy influences the development of non-union. We hypothesized that older age at the time of surgery, more severe radiographic acetabular dysplasia, obesity, and more medial superior ramus osteotomy would increase the risk of non-union at a minimum 1-year radiographic follow-up.

## PATIENTS AND METHODS

We retrospectively identified 320 patients who underwent a PAO to treat hip dysplasia from January 2003 to May 2020 with a minimum 1-year clinical and radiographic follow-up. Demographic information including age, sex, and BMI at the time of surgery was recorded. Four hips were excluded from the study for incomplete radiographs and clinical follow-up. The final cohort included 316 hips in 246 patients, 81.7% (*n* = 201) were women and the median [interquartile range (IQR)] age at the time of surgery was 23.9 (18.0–36.0) years (Table I).

**Table I.** Demographics with pre- and post-operative radiographic measurements in the entire cohort, hips with united superior ramus and hips with non-united superior ramus

	*All hips* *(n = 246 subjects/316 hips)*	*Superior ramus healed* *(n = 220 subjects/287 hips)*	*Superior ramus non-union* *(n = 26 subjects/29 hips)*	*P-value*
Demographics				
Age (years)^a^				
Subject level	25.0 (18.0–36.0)	23.0 (18.0–35.0)	36.5 (27.0–42.0)	<0.0001
Hip level	23.9 (18.0–36.0)	23.0 (18.0–35.0)	35.0 (26.0–40.0)	0.0010
BMI (kg/m^2^)^a^				
Subject level	25.5 (22.3–30.9)	25.0 (22.1–30.3)	29.7 (24.4–32.5)	0.0398
Hip level	25.5 (22.2–31.2)	25.2 (22.1–31.0)	29.1 (22.5–31.7)	0.0697
Female^b^				
Subject level	201 (81.7%)	180 (81.8%)	21 (80.8%)	1.0000
Hip level	260 (82.3%)	236 (82.2%)	24 (82.8%)	0.9434
Pre-operative variables (hip level)				
LCEA^a^	16 (11–20)	16 (11–20)	10 (6–13)	<0.0001
LCEA < 15º^a^	135 (42.7%)	111 (36.7%)	24 (82.8%)	<0.0001
ACEA ^a^	16 (7–23)	17 (9–24)	5 (−1–18)	0.0054
Post-operative variables (hip level)				
LCEA^a^	35 (30–38)	35 (30–38)	34 (29–38)	0.4375
ACEA^a^	31.7 (25–39)	32 (25–39)	31 (20–37)	0.3890
Osteotomy characteristics				
AP pelvis radiograph^a^ Ilioischial line to intact superior ramus	16.0 (13.8–18.8)	15.8 (13.2–18.7)	18.1 (16.2–20.5)	0.0009
Pelvis CT reconstruction^a^ Medial wall of the acetabulum to the superior ramus osteotomy	18.5 (14.8–22.7)	18.3 (14.6–22.5)	21.1 (15.6–25.6)	0.0520

a Median (IQR),

b Number (percent), *P*-values are for comparisons between healed and non-united hips.

PAO was performed by two surgeons (T.O.M. and M.C.W.), using previously described techniques [20], with osteotomies in the same order: superior ramus, incomplete osteotomy of the ischium and supra-acetabular osteotomy followed by osteotomy along the posterior column. The superior ramus osteotomy was performed with a straight stiletto osteotome using a Homan retractor to medially displace the hip flexors. The incomplete ischial cut was performed using a curved chisel, and the supra-acetabular osteotomy was performed using an oscillating saw and a combination of straight and curved osteotomes to complete the osteotomy along the posterior column. The post-operative protocol included flat foot–touch weight-bearing for 6 weeks followed by progression to weight-bearing as tolerated.

**Fig. 1.**
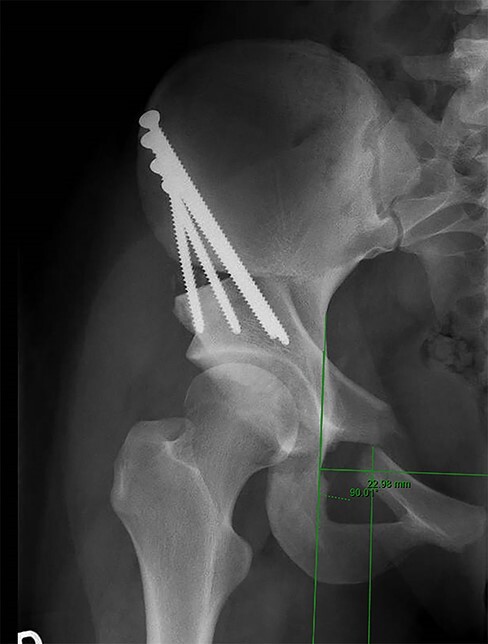
Method for the measurement of the distance of the superior ramus osteotomy from the ilioischial line on the AP pelvis radiograph: the first line is drawn along the ilioischial line. The second line is drawn parallel to ilioischial line to the superior tip of the intact medial ramus. The distance between the two lines is measured (22.98 mm in this case example).

**Fig. 2.**
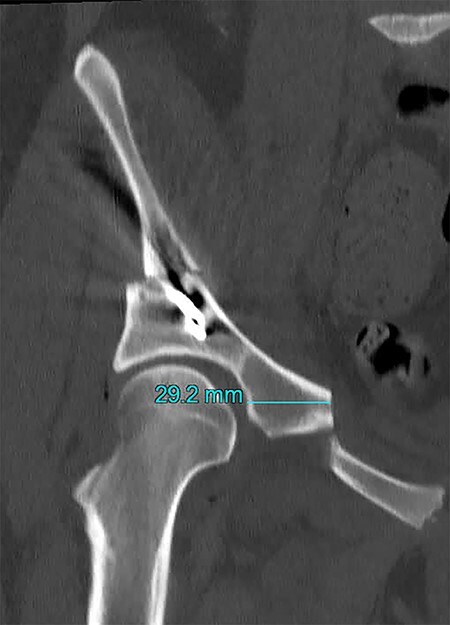
CT of the same patient in Fig. 1 in the immediate post-operative period showing the distance of the osteotomy site from the hip joint (29.2 mm).

Two authors (G.S. and J.H.) independently reviewed the medical records to retrospectively document patient demographics and clinical follow-up. All patients had a pre-operative standing anterior-posterior (AP) pelvis and false profile radiograph to measure the lateral center-edge angle (LCEA) of Wiberg [21] and the anterior center-edge angle (ACEA) of Lequesne [22], respectively. These radiographs were repeated at 6 and 12 months post-operatively. Measurements of LCEA and ACEA were performed on immediate pre-operative and 6 months post-operative radiographs by a single reviewer (M.C.W.). Assessment of superior ramus radiographic union was performed by two reviewers (G.S. and J.H.) on standing AP pelvis radiograph at a minimum of 12 months after PAO surgery. Non-union was defined as non-contiguous bony union with a persistent radiolucent line through the pubic ramus osteotomy. We defined a symptomatic non-union as the presence of radiographic non-union with persistent anterior or midline pelvis pain documented clinically. On the immediate post-operative AP pelvis radiograph, we measured the distance from the superior ramus osteotomy to the ilioischial line by first, drawing a line superimposed over the ilioischial line and then a line parallel to this from the tip of the superior-medial aspect of the osteotomy. We then measured the distance between these two lines (Fig. 1). Out of 316 hips, 260 (82%) hips underwent an immediate post-operative pelvis computed tomography (CT) after PAO. Using the post-operative CT scan, VITREA® (Canon Medical Informatics, Inc.) software allowed for the creation of oblique reconstructions to measure the distance from the medial wall of the acetabulum to the superior ramus osteotomy (Fig. 2). These measurements of the osteotomy position on the superior ramus and evaluation of superior ramus osteotomy union were repeated in 20 consecutive hips by 3 reviewers (M.C.W., J.D. and G.S.) to evaluate inter-rater reliability.

### Statistical analysis

Statistical analysis was performed using the SAS software version 9.4 (SAS institute Inc., Cary, NC, USA) and RStudio software version 4.0.2 (clusrank procedure [23]). The intraclass correlation coefficient (ICC) was used to describe the inter-rater reliability for osteotomy position on the superior ramus, while Kendall’s coefficient of concordance was used to describe the inter-rater reliability for superior ramus osteotomy union. Participant and hip characteristics were described overall and by the incidence of non-union. Categorical variables were described as frequency (percentage), and chi-square tests were used for non-union group comparisons. All continuous variables were reported as median (IQR) due to non-normal distributions, and the Wilcoxon rank-sum test for clustered data was used for non-union group comparisons. Logistic regression with generalized estimating equations to account for correlation between hips in participants with bilateral osteotomies was used to model the relationship between odds of non-union and potential predictor variables in univariate analyses. These variables included age, BMI, pre-operative and post-operative LCEA, distance of the superior ramus osteotomy from the ilioischial line and distance to the osteotomy from the joint line. The final multivariable model was selected using the quasi-information criterion and included variables significantly associated with odds of non-union. A *P*-value <0.05 was considered statistically significant.

## RESULTS

Twenty-nine of 316 hips (9.2%) were non-united at the superior ramus osteotomy on 12-month post-operative standing AP pelvis radiographs. Ten (3.2%) hips were documented as symptomatic with persistent anterior or midline pelvis pain. Six out of the 29 hips with superior ramus non-union developed an ischial stress fracture presumed to be a sequela of the superior ramus non-union. Five patients with superior ramus non-union and ischial stress fracture underwent a secondary operation for bone grafting and fixation of the superior ramus non-union (1.5%). One patient with superior ramus non-union and ischial stress fracture had resolution of ischium pain with presumed healing of the stress fracture, although the superior ramus remained radiographically non-united. This patient did not undergo a secondary surgery.

**Fig. 3.**
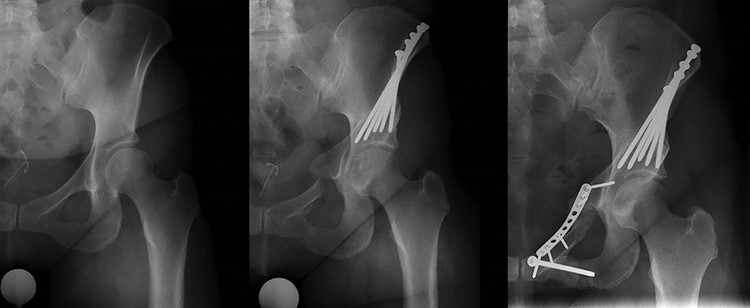
Pre-operative radiograph of a 38-year-old female with symptomatic hip dysplasia. Radiograph of the left hip at 1-year post-operative showing a non-union of the superior pubic ramus and a stress fracture of the inferior ramus. Radiograph 6 months after revision fixation and bone grafting of the non-union site and screw fixation of the inferior ramus showing a healed non-union with clinical resolution of symptoms. Reported an mHHS of 96 and an iHOT score of 84.5, two years after revision surgery.

Two patients with superior ramus osteotomy non-union underwent fixation and bone grafting through a modified Smith-Petersen approach (same approach used for the PAO surgery), and three patients underwent fixation and bone grafting through a modified Stoppa approach. Two of the patients who underwent bone grafting and fixation of the superior ramus non-union also underwent screw fixation of an ischial stress fracture (Fig. 3). Non-union fixation and bone grafting was performed a range of 14–18 months after index PAO surgery. All patients who underwent bone grafting and revision fixation radiographically healed the osteotomy, but three out of five patients had persistent dysfunction likely due to organ failure with osteoarthritis. Two patients with fixation and bone grafting of the superior ramus osteotomy underwent total hip arthroplasty (THA) at 1  and 6 years after the revision surgery. Another patient reported persistent, significant hip dysfunction but had not undergone THA 2 years after the revision surgery [modified Harris Hip Score (mHHS) 62 and International Hip Outcome Tool score (iHOT) 28.2]. Two patients reported good/excellent hip function after revision surgery (one patient 2 years after revision surgery reported an mHHS of 96 and an iHOT score of 84.5, and another patient 17 years after revision surgery reported an mHHS of 84 and an iHOT score of 97.5).

The distance measurement technique for the superior ramus osteotomy had excellent inter-rater reliability for a single reviewer [inter-rater ICC 0.97, 95% confidence interval (CI) = 0.94–0.98, *P* < 0.0001]. The Kendall’s coefficient for the presence of superior ramus non-union was 0.91 for a single reviewer.

The median (IQR) age of patients who healed the superior ramus was 23 (18–35) years compared to 35 (26–40) years for patients with non-union (*P* = 0.0010). Pre-operative median (IQR) LCEA was 16° (11°–20°) in hips with a healed superior ramus osteotomy compared to 10° (6°–13°) in hips with non-union (*P* < 0.0001). Additionally, the proportion of hips with pre-operative moderate-to-severe dysplasia (LCEA < 15°) was also higher in hips with non-union (83%) compared to hips with a healed osteotomy (37%) (*P* < 0.0001). The median (IQR) distance from the superior ramus osteotomy to the ilioischial line measured on AP pelvis radiograph was 15.8 mm (13.2–18.7) in hips with a healed osteotomy compared to 18.1 mm (16.2–20.5) hips with non-union (*P* = 0.0009). However, we found that medial distance of the osteotomy measured on CT [median (IQR) 18.3 mm (14.6–22.5) healed versus 21.0 mm (15.6–25.6) non-united, *P* = 0.0520] was not associated with non-union. BMI [median (IQR) 25.2 kg/m^2^ (22.1–31.0) for healed versus 29.1 kg/m^2^ (22.5–31.7) non-united, *P* = 0.0697] was not significantly different comparing hips with union and non-union.

The incidence of superior ramus non-union was similar comparing the two surgeons [Surgeon 1: 17/150 (11.3%) and Surgeon 2: 12/166 (7.2%), *P* = 0.207]. Surgeon 1 performed a significantly more medial superior ramus osteotomy compared to Surgeon 2 measured on the AP pelvis radiograph [Surgeon 1: mean (SD) 17.6 mm (4.2) and Surgeon 2: 14.8 mm (4.0), *P* < 0.0001] and measured on CT [Surgeon 1: mean (SD) 22.7 mm (3.9) and Surgeon 2: 15.2 mm (3.5), *P* < 0.0001].

In the multivariable model, we found that patients with versus without moderate-to-severe dysplasia (LCEA < 15°, OR 5.95, SE 3.32, 95% CI 1.99–17.79, *P* = 0.0014), increased age (5-year increase in age, OR 1.29, SE 3.32, 95% CI 1.05–1.60, *P* = 0.0180), and distance from the ilioischial line to the superior ramus osteotomy on AP pelvis radiograph (each 3-mm increase, OR 1.67, SE 0.22, 95% CI 1.29–2.18, *P* = 0.0001) were at a higher risk of non-union at 1 year (Table II).

**Table II.** Multivariate analysis of risk factors associated with non-union of the superior pubic ramus 12 months after PAO surgery

*Characteristics*	*OR (95% CI)*	*P-value*
LCEA pre-operatively < 15°	5.95 (1.99–17.79)	0.0014
Age (each 5-year increase)	1.29 (1.05–1.60)	0.018
AP pelvis radiograph Ilioischial line to the intact superior ramus (each 3-mm increase)	1.67 (1.29–2.18)	0.0001

## DISCUSSION

Non-union of the superior pubic ramus is a recognized, but under-reported complication when hip dysplasia is treated with PAO [12–14]. Although multiple studies report the prevalence of non-union, there are few that elucidate the risk factors. This retrospective study found a 9.2% incidence of non-union at the superior ramus osteotomy in 316 hips that underwent PAO. Increased age, increasing severity of dysplasia and the distance of the superior ramus osteotomy from the ilioischial line on the AP pelvis radiograph are independently associated with non-union 1 year post-operatively. The medial location of the osteotomy on AP pelvis radiograph was associated with non-union, but a comparable measurement on immediate post-operative CT was not significantly associated with non-union, indicating that CT is not necessary to assess risk of this complication. Quantifying the risk of superior ramus non-union after PAO allows a surgeon to identify high-risk patients so that interventions such as vitamin D optimization, nutrition supplementation and supplemental fixation/bone grafting may be implemented in high-risk patients with the goal of reducing this complication.

The incidence of superior ramus non-union of 9.2% is concordant with the reported rates of non-union in previous studies by Selberg *et al.* [13] (4.1%), Peters *et al.* [11] (12%) and Biedermann *et al.* [24] (13%). We also found a 2% (6/316) incidence of ischial stress fracture which is similar to other studies by Espinosa *et al.* [18] (0.5%), Peters *et al.* [11] (1.2%), Tsuboi *et al.* [25] (2.9%) and Hamai *et al.* [17] (4.7%). All cases of ischial stress fracture in our series were associated superior ramus non-union. Two of the five patients who underwent a procedure for fixation of the non-union also had screw fixation of the ischial stress fracture with subsequent healing of the stress fracture. In the native pelvis, higher forces are transmitted through the superior pubic ramus compared to the inferior pubic ramus [12]. This is altered by superior ramus non-union, and the additional stress can result in an ischial stress fracture.

**Fig. 4.**
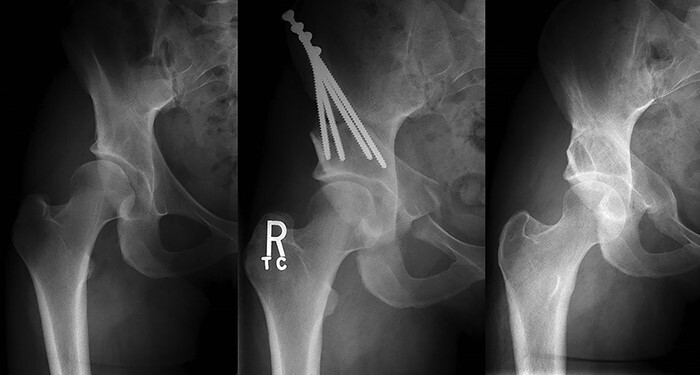
Pre-operative standing AP pelvis of a 24-year-old female who underwent PAO for hip dysplasia with subsequent 3-week post-operative standing AP pelvis radiograph and 1-year follow-up standing AP pelvis radiograph demonstrating the significant ability to heal a medial superior ramus osteotomy with significant displacement in a young, healthy individual.

Our study found that older age and more severe dysplasia independently predicted superior ramus osteotomy non-union after PAO. A recent study reporting the incidence of all types of non-union after PAO found that non-union was much more common 6 months after surgery (55%) [13]. Factors that predicted non-union at 6 months included age, obesity and severity of dysplasia (measured with LCEA). By 12 months after surgery, the incidence of non-union was down to 8% indicating that healing progresses >6 months after surgery. At 12-month follow-up, only age was an independent predictor of non-union. In our series with 12-month follow-up, the median age of patients who developed non-union was 35 years compared to 23 years in patients who healed the osteotomy (*P* = 0.0010). Our study also confirmed that more severe dysplasia (also quantified with LCEA) increased the risk for development of a superior ramus non-union (pre-operative LCEA median 16° healed versus 10° non-united, *P* < 0.0001). This is not surprising given the large displacement of the ramus that is required to achieve adequate coverage in severely dysplastic hips. Although it may seem inevitable that the ramus develops onto non-union in some cases with a large correction, the ramus appears to be capable of bridging significant gaps in young patients (Fig. 4). In other literature, higher BMI was found to be a risk factor for the surgical complications [15, 16], including non-union. Novias *et al.* [16] reported 7 of 215 PAO surgeries with non-union in the non-obese cohort compared to 5 of 65 cases of non-union in the obese cohort. We did not find increased BMI to be significantly associated with superior ramus non-union in our series. However, the median BMI of patients who went on to develop a non-union was 29.1 kg/m^2^ compared to 25.1 kg/m^2^ (*P* = 0.0697) for patients who healed the osteotomy. Increased BMI does put the patient at an increased risk for complications and worse post-operative outcomes as demonstrated in previous studies [15, 16]. Hence, it is prudent to counsel patients who are older, have a higher BMI and have severe dysplasia that they are at increased risk for complications with PAO, such as a non-union.

Previous studies have postulated that a more medial superior ramus osteotomy has a higher risk for non-union as the medial superior ramus bone is narrower with less contact area and requires more displacement for adequate correction [12]. Our study found a significant difference in the distance measured from the ilioischial line to the superior ramus osteotomy on standing AP pelvis between united and non-united osteotomies (15.8 mm compared to 18.8 mm for a hip with non-union, *P* = 0.0009). When a surgeon performs a PAO for a patient with other known risk factors such as older age and moderate-to-severe dysplasia, a more lateral superior ramus osteotomy may be beneficial for healing. This finding is concordant with another study by Matsunaga *et al*., who found that a gap >5.1 mm at the superior ramus osteotomy was an independent risk factor for a non-union [14]. On CT, we did not find that union was associated with medial distance of the osteotomy from the medial wall of the acetabulum. Although the difference was not significant, the median distance of the osteotomy from the joint in the healed group was 18.3 compared to 21.1 mm for the non-union group, which approached statistical significance (*P* = 0.052). These radiographic measurements indicate that location of the superior ramus osteotomy is important for healing and should be considered during PAO surgery.

Although fixation and bone grafting of the superior ramus non-union resulted in radiographic union in all revision cases, three out of five hips had persistent dysfunction (two converted to THA and one had significant impairment indicated by patient-reported outcomes). Failure of these hips was attributed to osteoarthritis progression. When considering revision surgery for superior ramus non-union, the status of osteoarthritis progression should be carefully assessed. Diagnostic intra-articular hip joint injection may be considered to determine if the source of persistent pain originates from the non-union or the hip joint. Regardless the patient should be informed of concerns about persistent hip dysfunction despite intervention.

Fixation of the ischium stress fracture was performed in two revision surgeries. Although this is technically achievable with a more medial ischial stress fracture as shown in Fig. 3, this may not be necessary as union of the superior ramus indirectly adds stability for the ischial stress fracture to heal.

### Limitations

This is a retrospective single-center study of two-surgeon’s practice over 17 years. These findings may not be broadly applicable to other centers and surgeons. Additionally, since this study included cases performed early in the experience by each surgeon, the frequency of the complication may be overestimated because some surgeries were performed early in the learning curve. Also, the sample of hips with a non-union of the superior pubic ramus is small (29) in relation to the study population (316), and hence, this may limit our ability to identify further risk factors. Finally, a significant proportion did not have a post-operative CT (56 of 316 hips). This may have limited our ability to find statistical significance with respect to non-union rates and the CT measurement.

### Conclusion

We found that non-union of the superior pubic ramus is a common complication following PAO and is associated with several independent risk factors; older age at time of surgery, moderate-to-severe dysplasia and medial position of the superior ramus osteotomy. This study will allow surgeons to quantify risks of surgery and to help devise strategies to reduce complications pre-operatively including optimization of bone density, vitamin D status, nutrition and weight loss. Intra-operatively, surgeons may choose to augment healing with a bone graft and/or additional fixation, lateralize the superior ramus osteotomy and prolong the duration of flat foot–touch weight-bearing in high-risk patients.

## Data Availability

The data underlying this article cannot be shared publicly for the privacy of the individuals involved in the study. The data will be shared upon reasonable request to the corresponding author.

## LEVEL OF EVIDENCE

Level IV.
